# Supplementary material for: High Diversity of Microcystin Chemotypes within a Summer Bloom of the Cyanobacterium Microcystis botrys
Source: Toxins (Basel). 2019 Dec 1;11(12):698. doi: 10.3390/toxins11120698 (PMC6950303; doi:10.3390/toxins11120698)
Supplement: Supplementary file 1 [file toxins-11-00698-s001.pdf]

Supplementary Materials: High Diversity of Microcystin Chemotypes within a Summer Bloom of the Cyanobacterium *Microcystis botrys*

Emma Johansson, Catherine Legrand, Caroline Björnerås, Anna Godhe, Hanna Mazur-Marzec, Torbjörn Säll and Karin Rengefors

Table S1. Mean microcystin content (ng mg^{-1} of dry weight) plus standard deviation of microcystin variants with standards.

Strain prefix	Sampling Date	MC-LR	MC-RR	[Asp^3, Dhb^7] MC-LR	[Dha^7]MC-RR	MC-YR
S1	2014-06-30	472.7 ± 82.3	117.0 ± 6.6	158.7 ± 9.2	135.7 ± 26.6	612.4 ± 87.6
S2	2014-07-14		38.0 ± 2.8			
S3	2014-08-03		36.3 ± 46.7			177.7
S4	2014-08-25	401.9 ± 151.2	73.6 ± 38.8	178.3 ± 34.5	101.7 ± 34.2	213.3 ± 133.7
S5	2014-09-08	426.9 ± 65.2	74.4 ± 33.8	116.0 ± 26.7	82.7 ± 43.4	165.1 ± 132.4

Table S2. Water chemistry data from Lake Vomb, sampled by the Vomb Water Works. Note that sampling dates are different from *Microcystis* colony sampling.

Sampling Date	Turbidity (FNU)	pH	Conductivity (mS m^{-1})	Ammonium (µmol 1^{-1})	Nitrite (µmol 1^{-1})	Nitrate (µmol 1^{-1})	Phosphate-P (µg P l^{-1})	Phosphate (µmol l^{-1})	Fe (mg l^{-1})	N:P (mol:mol)
May 12	15	8	45	5	1	161	6	0.19	0.01	864
June 9	1.2	8	45	2.78	1.2	105	<5	<0.16	<0.01	675
July 21	5.7	9	40	1.11	0.8	<14	13	0.42	0.01	39.1
Aug 18	8.2	8	38	1.67	0.2	<14	68	2.2	0.03	7.5
Sep 15	5.2	9	36	<0.55	0.2	<14	56	1.8	0.01	8.4

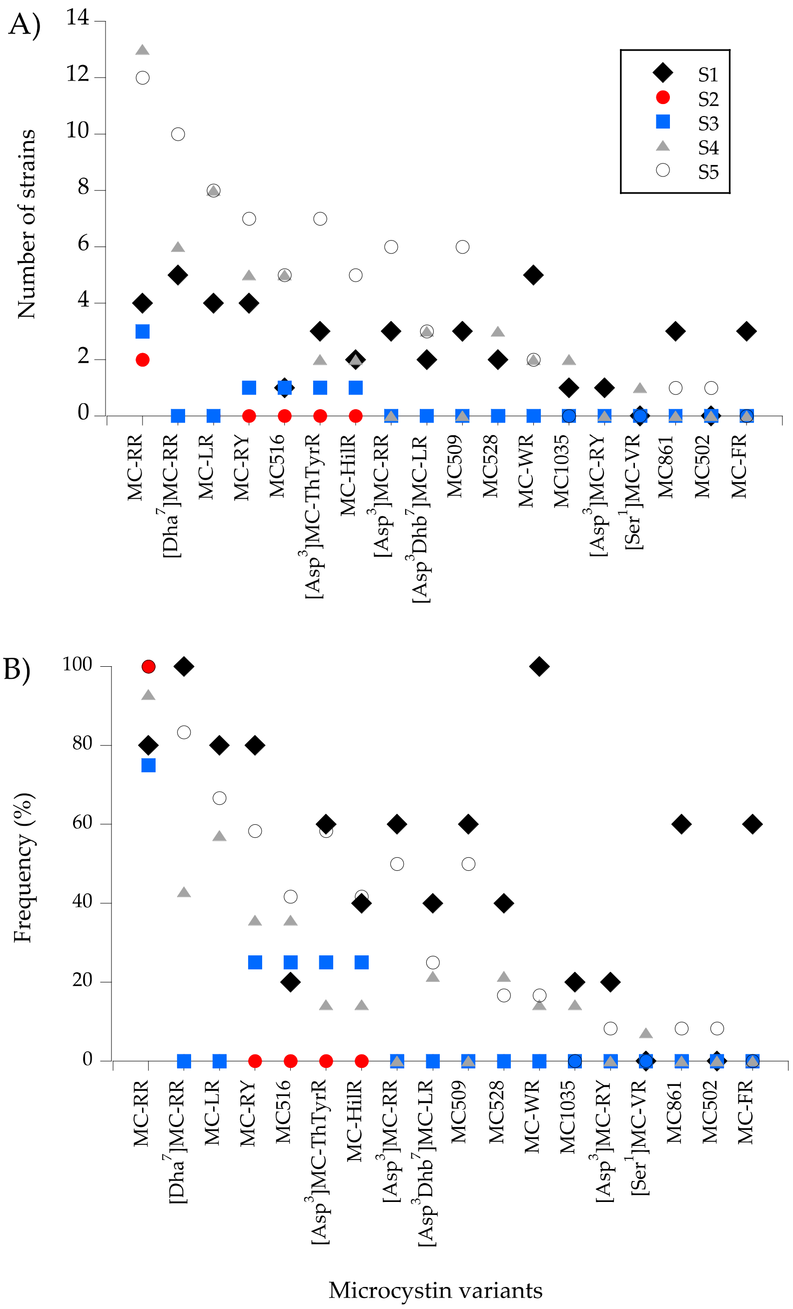

Figure S1: Prevalence of the 18 microcystin variants in sampled M. botrys strains, obtained by tandem mass spectrometry LC-MS/MS. (A) shows the absolute numbers of strains producing each microcystin variant within sampling occasions (S1-S5). (B) shows the frequencies (%) of strains producing each microcystin variant, within sampling occasions (S1-S5). Symbols denote sampling occasion: S1 = black diamonds, S2 = red circles, S3 = blue squares, S4 = grey triangles, S5 = open circles.

Figure S1. Prevalence of the 18 microcystin variants in sampled M. botrys strains, obtained by tandem mass spectrometry LC-MS/MS. (**A**) shows the absolute numbers of strains producing each microcystin variant within sampling occasions (S1–S5). (**B**) shows the frequencies (%) of strains producing each microcystin variant, within sampling occasions (S1–S5). Symbols denote sampling occasion: S1 = black diamonds, S2 = red circles, S3 = blue squares, S4 = grey triangles, S5 = open circles.

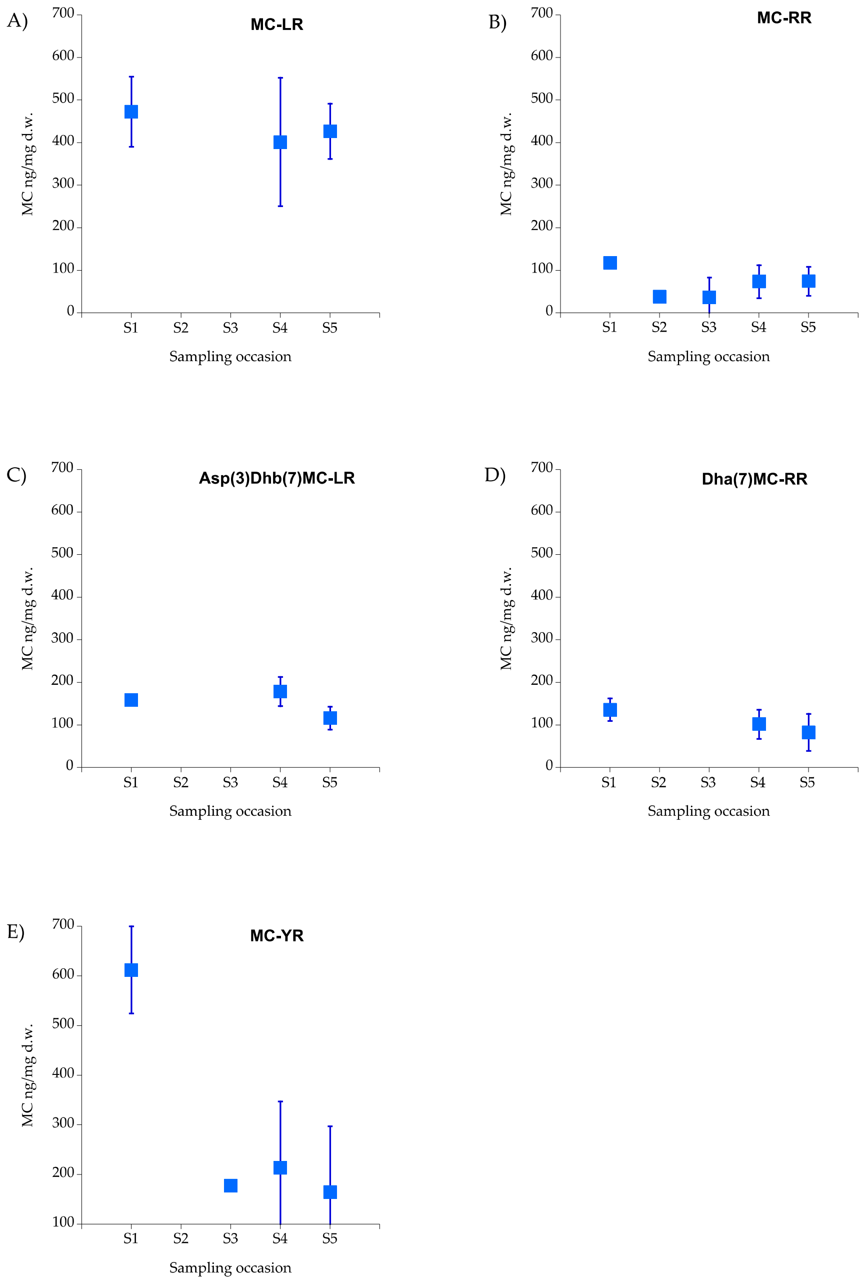

Figure S2. Microcystin content (MC ng mg-1 dry weight) for sampled M. botrys strains sampled at S1-S5, and analyzed using tandem mass-spectrometry LC-MS/MS. Mean values are shown for MCs where standards were available. Error bars shows standard deviations. (**A**) MC-LR, (**B**) MC-RR, (**C**) [Asp3, Dhb7]MC-LR, (**D**) [Dha7]MC-RR, (**E**) MC-YR.
